# Prevalence of ocular findings regardless of visual acuity status in older adults from the Brazilian Amazon Region

**DOI:** 10.1038/s41598-021-03172-4

**Published:** 2021-12-09

**Authors:** Arthur G. Fernandes, Adriana Berezovsky, Sung E. S. Watanabe, Márcia R. K. H. Mitsuhiro, Marcela C. Cypel, Nívea N. Ferraz, João M. Furtado, Paula Y. Sacai, Sergio Muñoz, Cristina C. Cunha, Galton C. Vasconcelos, Paulo H. A. Morales, Marcos J. Cohen, Jacob M. Cohen, Mauro Campos, Rubens Belfort, Solange R. Salomão

**Affiliations:** 1 Departamento de Oftalmologia E Ciências Visuais, Escola Paulista de Medicina, Universidade Federal de São Paulo–UNIFESP, R. Botucatu 821, São Paulo, SP CEP 04023-062 Brazil; 2 Departamento de Oftalmologia, Otorrinolaringologia E Cirurgia de Cabeça E Pescoço, Faculdade de Medicina de Ribeirão Preto da Universidade de São Paulo, Ribeirão Preto, SP Brazil; 3 Departamento de Salud Publica, Universidad de La Frontera, Temuco, Chile; 4 Faculdade de Medicina da Universidade Federal do Pará–UFPA, Belém, PA Brazil; 5 Departamento de Oftalmologia E Otorrinolaringologia, Faculdade de Medicina da, Universidade Federal de Minas Gerais–UFMG, Belo Horizonte, MG Brazil; 6 Divisão de Oftalmologia, Departamento de Cirurgia, Faculdade de Medicina da, Universidade Federal do Amazonas–UFAM, Manaus, AM Brazil

**Keywords:** Eye diseases, Epidemiology

## Abstract

Recently, it has been recommended that population-based studies report not only frequencies of vision impairment and blindness but also any ocular abnormalities that might lead an individual to seek for eyecare services. The current study aimed to determine prevalence of ocular findings regardless of visual acuity (VA) status in older adults from the Brazilian Amazon Region. Disturbances were grouped into: Eyelids; Anterior Segment; Posterior Segment; Increased intraocular pressure; and Overall Globe. The presence of an ocular finding was considered positive when any abnormality was noted, regardless of VA. Refractive errors were not considered. A total 2384 eligible persons were enumerated and 2041 (85.6%) examined. The prevalence of ocular disturbances in either eye was 87.0% and was associated with male gender, older age, lower education, and rural residence. Overall, main findings were pterygium, cataract, and pinguecula, occurring in 58.8%, 45.4% and 17.4%, respectively. Among individuals with 20/20 VA in both eyes, the most frequent findings were pterygium, pinguecula, and glaucoma cupping, occurring in 47.4%, 31.2% and 6.5%, respectively. The high prevalence of ocular findings observed in this population reinforces that different conditions might not immediately decrease VA but can indicate risk and/or discomfort symptoms and should be considered when planning public health ophthalmic services.

## Introduction

Blindness and visual impairment are important public health issues that affect approximately 596 million people worldwide^1^. Population-based studies on ocular epidemiology are usually devoted to determine the main causes of blindness and visual impairment obtained through abbreviated ophthalmic exam protocols in their vast majority^2–4^. However, there is scarcity of population-based cross-sectional surveys depicting the magnitude of ocular diseases among older adults except a few studies performed in African and Asian countries^5–9^.

The prevalence of non-vision impairing conditions (NVIC) is important from a public health perspective providing basis of making decisions related to training health personnel about common ocular conditions^5^. Data on the overall prevalence of ocular morbidity has usually not been included despite the fact that these conditions constitute the majority of consultations in eye clinics, avoiding proper planning of ophthalmic services, mainly in developing countries^8^. A study in Pakistan found prevalence of NVIC to be 14.6% after excluding presbyopia^9^. In a population-based survey in Kenya, one in each six participants showed an ocular condition at least in one eye, with diseases affecting the lens and conjunctiva among the most frequent^10^.

The Brazilian Amazon Region Eye Survey (BARES) is a population-based study of visual impairment and blindness among adults 45 years and older residing in both urban and rural areas in the city of Parintins^11^. Several epidemiologic aspects from BARES had been previously reported: the prevalence and causes of blindness and visual impairment for distance^12^ and near^13^; the impact of refractive correction on visual acuity^14^; the prevalence, visual outcomes and complications of cataract surgery^15^; and the prevalence of pterygium^16^.

The purpose of this study was to determine the prevalence of ocular findings in older adults from BARES, regardless of visual acuity status, detected after a comprehensive eye exam performed by experienced ophthalmologists.

## Methods

### Study population

The Brazilian Amazon Region Eye Survey (BARES) is a population-based, cross-sectional epidemiological study designed to examine the prevalence and causes of vision impairment and blindness in a non-institutionalized sample of older Brazilian Amazonians.

Parintins is a 102000 inhabitants city in the centre of the Brazilian Amazon Region on the sides of the Amazon River. The study population consisted of residents 45 years or older, living in 20 clusters randomly selected based on cluster sampling (14 urban and 6 rural) from the 2010 Census data. Further details on sampling plan, and baseline demographic data have been reported elsewhere^11^. In brief, a door-to-door enumeration was performed and eligible subjects were informed about the study and invited to a clinical ophthalmic examination.

### Ethical approval and informed consent

The institutional review board/ethics committees both from Universidade Federal de São Paulo (UNIFESP) and from Universidade Federal do Amazonas (UFAM) approved the study protocol. The study was carried out in accordance to the tenets of the Declaration of Helsinki. Written informed consent was obtained from all participants after explanation of the nature and possible consequences of the study. In cases of illiterate individuals, the informed consent was obtained from their legally authorized representatives.

### Sociodemographic data

The enumeration form included household address, phone number, and a roster of those living in that home along with their gender, age and schooling. All eligible individuals (adults 45 years of age and older) were invited and scheduled for a detailed eye examination. Written informed consent was obtained at the examination site, followed by the eye examination.

### Clinical data

A comprehensive eye examination was conducted following a similar protocol used in an earlier study in Brazil, the São Paulo Eye Study (SPES)^17^. For participants who could not come to the clinic for the exam, an in-home eye exam was offered and it was performed with portable equipment.

Ophthalmic technologists measured from each eye presenting distance visual acuity (PVA), with spectacles if the participant presented with them followed by uncorrected distance visual acuity (UCVA), using retro-illuminated logarithm of the minimum angle of resolution tumbling E charts at 4 m distance. Best-corrected visual acuity (BCVA) was determined for each eye after auto-refraction followed by subjective refraction performed by an ophthalmologist.

The eye examination included anterior segment biomicroscopy, lens status assessment, intraocular pressure (IOP) measurement and fundus examination under dilation. Ocular findings were grouped into 5 categories: Eyelids; Anterior Segment; Posterior Segment; Increased IOP; and Overall Globe.

The presence of an ocular finding was considered positive when any abnormality was noted during the eye examination, regardless of the current visual acuity status and/or the potential visual impairment that could be caused by the abnormality. For the purposes of this study, presbyopia and refractive error were excluded.

### Statistical analysis

Statistical analyses were performed using Stata/SE Statistical Software, Release 14.0, 2015 (Stata Corp, College Station, Texas, USA). Frequency tables were used for descriptive analysis. The associations between categorical variables and co-variables were evaluated through Firth’s penalized likelihood logistic regression. Confidence intervals (CI) for regression odds ratios (OR) were calculated taking cluster sampling design effects into account. *P* values ≤ 0.05 were considered statistically significant.

## Results

Out of the 9931 residents from the selected clusters, 2384 (24.0%) were eligible for the study. Of these, 2041 completed an ophthalmic examination, representing a participation rate of 85.6% whereas 335 (14.1%) did not show up for the clinical examination and 8 (0.3%) refused to participate. Demographic characteristics of the study population were described in detail previously. Table 1 shows the distribution of sex, age, educational level and area of residence of enumerated and examined participants.

**Table 1:** Enumerated and examined population by sex, age, education, and area of residence.

	Enumerated	Examined	Percent examined
	*N* (%)	*N* (%)	*N* (%)
**Sex**			
Male	1198 (50.25)	1000 (49.00)	83.47
Female	1186 (49.75)	1041 (51.00)	87.77
**Age**			
45–54	936 (39.26)	790 (38.71)	84.4
55–64	753 (31.59)	646 (31.65)	85.79
65–74	395 (16.57)	351 (17.20)	88.86
75 or more	300 (12.58)	254 (12.44)	84.67
**Education**			
None	302 (12.67)	237 (11.61)	78.48
Less than Primary	631 (26.47)	546 (26.75)	86.53
Primary	662 (27.77)	577 (28.27)	87.16
Secondary	345 (14.47)	295 (14.45)	85.51
High School or higher	444 (18.62)	386 (18.91)	86.94
**Area of residence**			
Urban	1410 (59.14)	1180 (57.81)	83.69
Rural	974 (40.86)	861 (42.19)	88.4
**Total**	**2384 (100.00)**	**2041 (100.00)**	**85.61**

Examination response was associated with female gender (odds ratio [OR] 1.44; 95% confidence interval [CI] 1.06–1.94; *p* = 0.022), age between 65 and 74 years (OR 1.84; 95% CI 1.29–2.62; *p* = 0.002), and rural residency (OR 1.91; 95% CI 1.24–2.94; *p* = 0.006). Yet, higher education was associated with examination response for the categories “less than primary” (OR 2.16; 95% CI 1.15–4.04; *p* = 0.019), “primary” (OR 2.57; 95% CI 1.55–4.24; *p* = 0.001), “secondary” (OR 2.53; 95% CI 1.64–3.93; *p* < 0.001), and “high school or higher” (OR 2.91; 95% CI 1.58–5.35; *p* = 0.002).

A total of 1776 participants (87.02%; 95% CI 85.49–88.41%) showed ocular disturbances in either eye regardless of visual acuity status. Eyelid findings were observed in 76 individuals (3.72%), Anterior segment in 1715 individuals (84.03%), Posterior segment in 433 (21.22%), increased IOP in 49 (2.40%), and overall globe issues in 23 (1.13%). Table 2 shows the frequency of ocular finding in the different categories.

**Table 2:** Ocular findings per person by area of residence.

Ocular findings	Rural area	Urban area	All
	*N* (%)	*N* (%)	*N* (%)
**No abnormalities**	91 (10.57)	174 (14.75)	265 (12.98)
**Eyelids**			
Blepharitis	6 (0.70)	40 (3.39)	46 (2.25)
Structural abnormality^1^	17 (1.97)	14 (1.19)	31 (1.52)
**Anterior segment**			
Cornea			
Cornealscar/opacification	48 (5.57)	68 (5.76)	116 (5.68)
Keratitis	3 (0.35)	7 (0.59)	10 (0.49)
Conjunctiva			
Pterygium	548 (63.65)	651 (55.17)	1199 (58.75)
Pinguecula	173 (20.09)	182 (15.42)	355 (17.39)
Conjunctivitis	6 (0.70)	12 (1.02)	18 (0.88)
Iris			
Structural abnormality^2^	17 (1.97)	27 (2.29)	44 (2.16)
Absent puppilary response	18 (2.09)	21 (1.78)	39 (1.91)
Lens			
Cataract	379 (44.02)	547 (46.36)	926 (45.37)
Pseudoexfoliation	5 (0.58)	12 (1.02)	17 (0.83)
**Posterior Segment**			
Glaucoma cupping	80 (9.29)	100 (8.47)	180 (8.82)
Maculopathy^3^	40 (4.65)	85 (7.20)	125 (6.12)
Scar^4^	32 (3.72)	40 (3.39)	72 (3.53)
Age-related macular degeneration	22 (2.56)	20 (1.69)	42 (2.06)
Vascular retinopathy	15 (1.74)	20 (1.69)	35 (1.71)
Vitreous opacity	17 (1.97)	13 (1.10)	30 (1.47)
Diabetic retinopathy	9 (1.05)	13 (1.10)	22 (1.08)
Other optical atrophy	4 (0.46)	7 (0.59)	11 (0.54)
High myopia	2 (0.23)	6 (0.51)	8 (0.39)
Retina detachment	1 (0.12)	4 (0.34)	5 (0.24)
Retinitis pigmentosa	1 (0.12)	2 (0.17)	3 (0.15)
**IOP ≥ 21 mmHg**	5 (0.58)	44 (3.73)	49 (2.40)
**Globe**			
*Phthisis bulbi*	8 (0.93)	14 (1.19)	22 (1.08)
Absent/desorganized	0 (0.00	1 (0.08)	1 (0.05)
**Total**	**861 (100.00%)**	**1180 (100.00%)**	**2041 (100.00%)**

^1^Entropion, irregular margins, ptosis; ^2^Corectopy, iris atrophy, synechiae; ^3^Maculopathy, drusen, retinal pigment epithelium atrophy; ^4^chorioretinitis, peripapilary atrophy, congenital abnormalities.

*The totals for the cause specific prevalence exceed the Total because a person can present two or more different ocular conditions in either eye.

Overall, the main observed findings were pterygium, cataract, and pinguecula, occurring in 58.75%, 45.37% and 17.39%, respectively. Multiple logistic regressions were performed to investigate the association of each ocular finding outcomes with sex, age, education level and area of residence. Any disturbances in either eye was significantly associated with male gender (OR = 1.63; 95% CI 1.24–2.15; *p* = 0.001), older age (OR = 5.93; 95% CI 2.75–8.50; *p* < 0.001), lower education (OR = 3.42; 95% CI 1.56–7.51; *p* = 0.002), and marginally associated with rural residence (OR = 1.33; 95% CI 1.00–1.78; *p* = 0.052).

Male sex was associated with higher frequency of pterygium and glaucoma cupping when compared to female. Men were 1.63 times as likely to have pterygium than women (OR = 1.63; 95% CI 1.37–1.94; *p* = 0.001) and 1.71 times as likely to present glaucoma cupping (OR = 1.71; 95% CI 1.25–2.35; *p* = 0.001).

Individuals aged 75 years and more were more likely to show eyelid structural abnormalities (OR = 5.03; 95% CI 1.49–17.00; *p* = 0.009), corneal scar/opacification (OR = 6.14; 95% CI 3.35–11.28; *p* < 0.001), iris structural abnormalities (OR = 11.79; 95% CI 4.06–34.24; *p* < 0.001), absent pupillary response (OR = 8.02; 95% CI 2.70–23.82; *p* < 0.001), cataract (OR = 12.37; 95% CI 8.49–18.01; *p* < 0.001), glaucoma cupping (OR = 1.81; 95% CI 1.59–3.95; *p* = 0.033), maculopathy (OR = 4.59; 95% CI 2.48–8.51; *p* < 0.001), age-related macular degeneration (OR = 19.86; 95% CI 5.31–74.25; *p* < 0.001), increased IOP (OR 3.90; 95% CI 1.58–9.64; *p* = 0.003), and globe phthisis bulbi (OR = 4.26; 95% CI 1.26–14.47; *p* = 0.020) when compared to those aged 45–54 years old.

Individuals with lower education were more likely to present pterygium (OR = 1.58; 95% CI 1.08–2.30; *p* = 0.018) and cataract (OR = 4.14; 95% CI 2.62–6.54; *p* < 0.001) when compared to individuals with higher education.

Rural area residents were more likely to have blepharitis (OR = 2.24; 95% CI 1.46–3.44; *p* < 0.001), cataract (OR = 1.37; 95% CI 1.09–1.73; *p* = 0.007), maculopathy (OR = 1.25; 95% CI 1.02–1.53; *p* < 0.001), and increased IOP (OR 9.71; 95% CI 3.56–26.48; *p* < 0.001) and less likely to show pinguecula (OR = 0.83; 95%I: 0.73–0.93; *p* = 0.002) when compared to urban areas residents.

Reliable visual acuity measurements were obtained from 2025 participants. Uncorrected visual acuity (UCVA) 20/20 in both eyes was observed in 215 (10.62%), UCVA 20/25 or better in both eyes were observed in 414 (20.44%) and UCVA 20/32 or better in both eyes were observed in 647 (31.95%).

Individuals with UCVA 20/32 or better in both eyes were mostly males (53.79%), aged 45 to 54 years old (66.92%), with primary level education (30.29%) and urban residents (61.67%). Table 3 shows the distribution of ocular findings in the participants, according to the UCVA.

**Table 3:** Ocular findings in either eye according to the uncorrected visual acuity by area of residence.

Ocular findings	Rural area			Urban area			All		
	UCVA 20/20 OU	UCVA ≥ 20/25 OU	UCVA ≥ 20/32 OU	UCVA 20/20 OU	UCVA ≥ 20/25 OU	UCVA ≥ 20/32 OU	UCVA 20/20 OU	UCVA ≥ 20/25 OU	UCVA ≥ 20/32 OU
	*N* (%)	*N* (%)	*N* (%)	*N* (%)	*N* (%)	*N* (%)	*N* (%)	*N* (%)	*N* (%)
**No abnormalities**	18 (20.93)	33 (20.89)	48 (19.35)	38 (29.46)	61 (23.83)	90 (25.56)	56 (26.05)	94 (22.70)	138 (21.33)
**Eyelids**									
Blepharitis	1 (1.16)	1 (0.63)	2 (0.81)	6 (2.79)	11 (4.30)	16 (4.01)	6 (2.79)	12 (2.90)	18 (2.78)
Structural abnormality^1^	0 (0.00)	1 (0.63)	1 (0.40)	1 (0.78)	1 (0.39)	2 (0.50)	1 (0.47)	1 (0.24)	3 (0.46)
**Anterior segment**									
Cornea									
Corneal scar/opacification	1 (1.16)	4 (2.53)	6 (2.42)	1 (0.78)	4 (1.56)	7 (1.75)	2 (0.93)	8 (1.93)	13 (2.01)
Keratitis	0 (0.00)	0 (0.00)	0 (0.00)	1 (0.78)	1 (0.39)	1 (0.25)	1 (0.47)	1 (0.24)	1 (0.15)
Conjunctiva									
Pterygium	46 (53.49)	93 (58.86)	145 (58.47)	56 (43.41)	128 (50.00)	145 (58.47)	102 (47.44)	221 (53.38)	348 (53.79)
Pinguecula	32 (37.21)	41 (25.95)	64 (25.81)	35 (27.13)	65 (25.39)	94 (23.56)	67 (31.16)	106 (25.60)	158 (24.42)
Conjunctivitis	0 (0.00)	0 (0.00)	0 (0.00)	2 (1.55)	2 (0.78)	3 (0.75)	2 (0.93)	2 (0.48)	3 (0.46)
Iris									
Structural abnormality^2^	0 (0.00)	1 (0.63)	1 (0.40)	0 (0.00)	1 (0.39)	3 (0.75)	0 (0.00)	2 (0.48)	4 (0.62)
Lens									
Cataract	2 (2.33)	11 (6.96)	28 (11.29)	11 (8.53)	38 (14.84)	84 (21.05)	13 (6.05)	49 (11.84)	112 (17.31)
Pseudoexfoliation	0 (0.00)	1 (0.63)	1 (0.40)	0 (0.00)	0 (0.00)	0 (0.00)	0 (0.00)	1 (0.24)	1 (0.15)
**Posterior Segment**									
Glaucoma cupping	6 (6.98)	13 (8.23)	24 (9.68)	8 (6.20)	15 (5.86)	22 (5.51)	14 (6.51)	28 (6.76)	46 (7.11)
Maculopathy^3^	0 (0.00)	1 (0.63)	4 (1.61)	5 (3.88)	10 (3.91)	18 (4.51)	5 (2.33)	11 (2.66)	22 (3.40)
Scar^4^	3 (3.49)	4 (2.53)	7 (2.82)	3 (2.33)	9 (3.52)	13 (3.26)	6 (2.79)	13 (3.14)	20 (3.09)
Vascular retinopathy	0 (0.00)	0 (0.00)	1 (0.40)	4 (3.10)	6 (2.34)	7 (1.75)	4 (1.86)	6 (1.45)	8 (1.24)
ARMD	0 (0.00)	0 (0.00)	0 (0.00)	1 (0.78)	1 (0.39)	1 (0.25)	1 (0.47)	1 (0.24)	1 (0.15)
Vitreous opacity	0 (0.00)	1 (0.63)	2 (0.81)	0 (0.00)	1 (0.39)	2 (0.50)	0 (0.00)	2 (0.48)	4 (0.62)
Diabetes retinopathy	0 (0.00)	0 (0.00)	2 (0.81)	1 (0.78)	3 (1.17)	4 (1.00)	1 (0.47)	3 (0.72)	6 (0.93)
Retina detachment	0 (0.00)	0 (0.00)	0 (0.00)	0 (0.00)	0 (0.00)	1 (0.25)	0 (0.00)	0 (0.00)	1 (0.15)
**IO*P*** **≥** **21mmHg**	0 (0.00)	0 (0.00)	0 (0.00)	0 (0.00)	0 (0.00)	8 (2.01)	0 (0.00)	0 (0.00)	8 (1.24)
**Total**	**86 (100.00)**	**158 (100.00)**	**248 (100.00)**	**129 (100.00)**	**256 (100.00)**	**399 (100.00)**	**215 (100.00)**	**414 (100.00)**	**647 (100.00)**

^1^Entropion irregular margins, ptosis; ^2^Corectopy, iris atrophy, synechiae; ^3^Maculopathy, drusen, retinal pigment epithelium atrophy; ^4^corioretinitis, peripapilary atrophy, congenital abnormalities.

*The totals for the cause specific prevalence exceed the Total because a person can present two or more different ocular conditions in either eye.

Absence of any ocular abnormalities in both eyes was detected in 26.05%, 22.70% and 21.33% of those participants with UCVA of 20/20 in both eyes, UCVA of 20/25 or better in both eyes and 20/32 or better in both eyes, respectively.

When considering individuals with 20/20 vision in both eyes, the most frequently ocular findings were pterygium (47.44%), pinguecula (31.16%) and glaucoma cupping (6.51%). When including those with 20/25 or 20/32, cataract became the third main finding.

Cataract was observed in 112 participants (17.31%) among those not visually impaired (UCVA ≥ 20/32 in both eyes), affecting 199 eyes. In these cases, the most frequent cataract classifications were nuclear (66.33%), cortical (15.58%), and subcapsular (9.55%). Yet, among those 647 participants not visually impaired, 29 individuals (4.48%) had undergone cataract surgery in either eye summing 52 pseudophakic eyes that also showed pterygium (19.23%), pinguecula (13.46%), and glaucoma cupping (9.61%) as the most frequent findings.

## Discussion

The current study brings data from a population-based study beyond the causes of visual impairment and blindness. A comprehensive ocular exam was performed and any ocular abnormality was noted, regardless of its potential to affect visual acuity. The protocol was carried out with methodological rigor and each evaluation was conducted and/or supervised by senior ophthalmologists^11^.

The majority of population-based studies report data on frequencies and causes of visual impairment and blindness as those are the main focus of public health policies worldwide focused on the elimination of avoidable blindness^18^. These studies, however, may overlook the burden of disease as they consider only visual impairment conditions and count only the better seeing eye visual acuity. Other than the cases of visual impairment and blindness, the need for an eye consultation can include conditions with the potential of causing visual impairment as glaucoma cupping and diabetic retinopathy and also conditions that are symptomatic but not likely to decrease visual acuity as conjunctiva disorders^19^.

Our findings showed a prevalence of any ocular abnormality of 87.0% (95% CI 78.7–92.4%) while a previous report from the same population showed a prevalence of any level of presenting visual impairment and blindness in the better seeing eye of 39.8% (37.7–42.0%)^12^. The discrepancy highlights the importance of considering non vision impairing conditions on planning eye care public health policies as most cases demanding an ophthalmic examination might not be those associated with visual impairment or blindness. A previous study performed in the UK with over 3 million people attending eye health consultations showed that 88.1% of cases were not associated with visual impairment complaint^20^. Similar results were recently observed in India and Kenya, with conditions not associated with visual acuity decrease accounting for 59% and 52%, respectively^19,21^.

In accordance to those previous studies, most cases in our sample affected the eye anterior segment, with pterygium and cataract as the most frequent abnormalities^10,19–21^. In terms of posterior segment, glaucoma cupping was the most observed finding. Eyelids, increased intraocular pressure and globe disorders were less frequent.

Pterygium was the most frequent finding affecting over half of the population. Its high prevalence may be associated to the region geographic location characterized by low latitude and high ultraviolet (UV) exposure. The population profile is also a determinant for the pterygium development so that people who have an outdoor lifestyle tends to be more likely to develop the disease as the direct UV exposure is reduced^16^. In that sense, pterygium presence was associated with male gender and lower education, also linked to the higher UV exposure as men and those with lower education are usually reported as outdoor workers. These assumptions, however, could not be properly evaluated in our study as there are no information regarding the occupation of the participants.

Cataract was the second main finding and showed to be associated with older age, lower education and rural residence which reflects both the natural history of disease and the limited access to treatment in this population. The lens is a structure formed by epithelial cells that constantly generates lens fibers throughout life and, differently than what occurs in the skin tissue, old lens fibers are not loss and so, as result of the aging process, the lens become more compact and thicker, leading to loss on its transparency and to the cataract formation^22–24^. As published before, the cataract surgical coverage in this population was 42.1% which explain the high frequency of participants presenting cataract in this analysis^15^.

When comparing the main findings from the total population versus participants without visual impairment, pterygium remained as the principal abnormality affecting 58.7% and 53.8% of the population, respectively. Cataract frequency, however, decreased from 45.4% in the total population to 17.3% in those not visually impaired. Pterygium impact on visual acuity in less usual and it is associated with high levels of astigmatism secondary to lesions greater than 3 mm^16^. Cataract, on the other hand, progressively decreases visual acuity as the intraocular lens looses its transparency. Among the not visually impaired eyes, the less common type of cataract was the posterior subcapsular cataract, a morphology typically associated with obscuring the eye’s nodal point resulting in central visual loss and visual acuity decrease^25,26^.

Glaucoma cupping was noted in 8.82% of the overall population and in 7.11% of those not visually impaired. Interestingly, the prevalence of glaucoma as cause of visual impairment and blindness accounted for only 1.83% of the population^12^. The estimated number of people living with glaucoma is substantially greater than the estimated number of people visually impaired by glaucoma because the central visual acuity of most affected patients is preserved^19^. Data restricted to visual impairment and blindness underestimate the need of eye care health services for patients with glaucoma and it should be considered when developing public health policies towards this disease. Moreover, glaucoma cupping might be an indicative of early-stage disease or high risk of disease development that could indeed cause vision impairment in later life in a person who will need ongoing care to prevent the disease development or progression. While our data showed association of glaucoma cupping with male gender and older age, there is no consensus in the literature with studies indicating significant effects only of aging^27^, only of male gender^28^, both^29^ or neither^30^. A detailed analysis of the optic nerve head topography would be indicated to a better understand of those variables’ effects.

Other ocular findings less threating to visual acuity but still symptomatic were noted in lower frequencies and might be associated to the population profile and orientation. Blepharitis, keratitis, and conjunctivitis are conditions reported to be associated with higher health literacy and better personal hygiene^31–34^. Corneal scar was present in 5.68% of the total population and in 2.01% of those not visually impaired, with the cases of the last group probably affecting the cornea periphery. Corneal scarring is often associated with work related trauma history and may indicate the lack of use of proper eye protection equipment^35,36^. Simple interventions towards population education for prevention of such conditions could minimize those cases.

Even among individuals with uncorrected visual acuity 20/20 in both eyes, almost 3/4 presented any ocular abnormality in either eye. Non-vision-impairing conditions summed most cases with pterygium and pinguecula as the most frequent, however, glaucoma cupping showed to be the third most frequent finding and, as mentioned, it needs to be evaluated closely.

A high prevalence of ocular findings regardless of visual acuity status was observed in this population, mainly affecting conjunctiva and lens. These findings reinforce that not only subjects with visual impairment and blindness are in need of eye health care. Different ocular findings might not immediately decrease visual acuity but can indicate risk of disease development and/or cause discomfort symptoms. In that sense, healthcare authorities and policy makers should evaluate ocular data beyond the visual acuity in order to provide access and proper facilities to guarantee the best ocular health for the population.
